# Mastering your fellowship

**DOI:** 10.4102/safp.v62i1.5233

**Published:** 2020-11-11

**Authors:** Mergan Naidoo, Klaus B. von Pressentin, Honey Mabuza, Tasleem Ras

**Affiliations:** 1 Department of Family Medicine, College of Health Sciences, University of KwaZulu-Natal, Durban, South Africa; 2 Division of Family Medicine, University of Cape Town, Cape Town, South Africa; 3 Department of Family Medicine and Primary Health Care, Sefako Makgatho Health Sciences University, Pretoria, South Africa

**Keywords:** Fellowship of the College of Family Physicians, FCFP (SA), examination preparation, family medicine registrars

## Abstract

The series, ‘Mastering your Fellowship’, provides examples of the question format encountered in the written and clinical examinations, Part A of the Fellowship of the College of Family Physicians, South Africa (FCFP [SA]) examination. The series is aimed at helping family medicine registrars prepare for this examination. Model answers are available online.

## Introduction

This section in the *South African Family Practice* journal is aimed at helping registrars prepare for the Fellowship of the College of Family Physicians, South Africa (FCFP [SA]) Final Part A examination and will provide examples of the question formats encountered in the written examination: multiple choice question (MCQ) in the form of single best answer (SBA – Type A) and/or extended matching question (EMQ – Type R); short answer question (SAQ), questions based on the critical reading of a journal (evidence-based medicine) and an example of an objectively structured clinical examination (OSCE) question. Each of these question types is presented based on the College of Family Physicians blueprint and the key learning outcomes of the FCFP programme. The MCQs will be based on the 10 clinical domains of family medicine, the modified essay questions (MEQs) will be aligned with the five national unit standards and the critical reading section will include evidence-based medicine and primary care research methods.

This month’s edition is based on unit standard 1 (critically review new evidence and apply the evidence in practice), unit standard 2 (evaluate and manage a patient according to the biopsychosocial approach) and unit standard 4 (facilitate the learning of others). The domains covered in this edition are otorhinolaryngology, ophthalmology and dermatology. We suggest that you attempt answering the questions (by yourself or with peers/supervisors) before finding the model answers online at: http://www.safpj.co.za/.

For guidelines on the Fellowship examination, visit the Colleges of Medicine’s website: https://www.cmsa.co.za/view_exam.aspx?QualificationID=9.

We are keen to hear about how this series is assisting registrars and their supervisors in preparing for the FCFP (SA) examination. Please email us your feedback and suggestions.

### Multiple choice question: Single best answer

A 52-year-old man complains of dizziness on awakening. All his vital signs are normal and the patient is counselled for a manoeuvre. On rotating the neck 45° and quickly lowering the patient over the edge of the bed you notice nystagmus, which lasts 30 s. All other clinical findings and bedside investigations are normal. The most appropriate next step is to:

- perform the Epley manoeuvre on the patient
- prescribe an antihistamine
- reassurance on the benign nature of the condition
- refer the patient to an ENT surgeon
- request further investigations.

*Answer*: (a)

The clinical scenario described here is a classic presentation of a patient with benign paroxysmal positional vertigo (BPPV). This is a common condition causing dizziness and usually occurs suddenly. It characteristically presents with vertigo and nystagmus associated with positional changes. Symptoms may last from days to weeks to month. The Dix–Hallpike manoeuvre described in this scenario is the typical clinical test for BPPV and positive findings included nystagmus for a limited duration, and this is considered diagnostic. A negative test result does not rule out BPPV.

The Dix–Hallpike manoeuvre is performed by quickly moving the patient from a sitting position usually with the arms folded to the supine position with the head turned 45° to the right, allowing for extension of the neck over the bed. Wait for 30 s and then return the patient to the sitting position whilst observing for nystagmus. The procedure should then be done for the left side.

**FIGURE 1:**
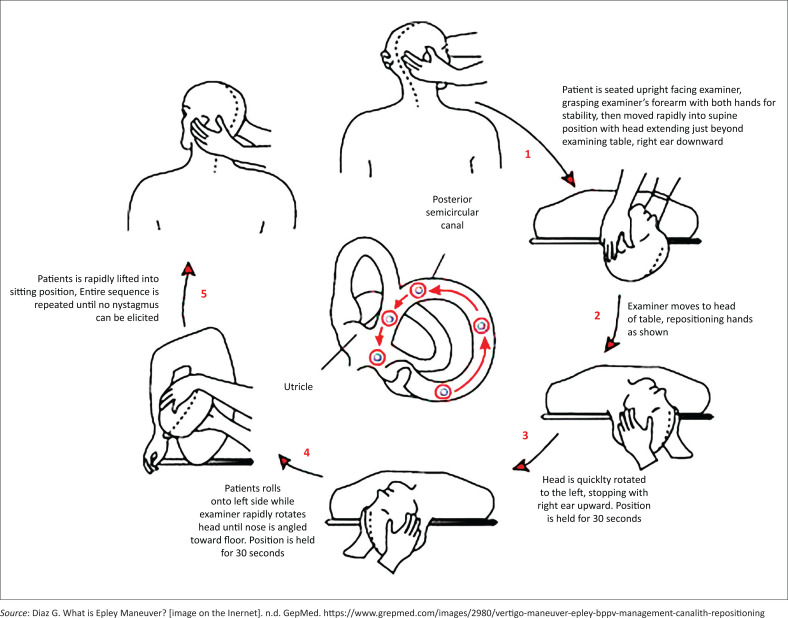
Pictorial depiction of the Epley manoeuvre.

Various treatment options exist, which include reassurance and vestibule suppressant medication, but the benefit of canalith repositioning such as the Epley manoeuvre is the treatment modality of choice and can cure the patient with one of two treatments in the consulting room. The procedure is depicted in Figure 1. Surgery is usually reserved for those in whom the canalith repositioning procedure fails and such patients will need to be referred to the ear, nose and throat (ENT) surgeon.

Epley suggested that symptoms of BPPV were because of free-moving densities (canaliths) in the posterior semicircular canals (PSC). When the head is upright, the particles sit in the PSC at the most gravity-dependent position. When the head is tilted back supine, the particles are rotated up approximately 90° along the arc of the PSC. After a momentary (inertial) lag, gravity pulls the particles down the arc. This causes the endolymph to flow away from the ampulla and causes the cupula to be deflected. The cupular deflection produces nystagmus. Reversal of the rotation (sitting back up) causes reversal of the cupular deflection and thus dizziness with nystagmus beating in the opposite direction.

There are many predisposing factors of BPPV that include trauma, otitis media, vestibular neuritis, Ménière’s disease, otosclerosis, sudden sensorineural hearing loss and central nervous system disease. The cause is however thought to be idiopathic. No further investigations are generally advocated in the presence of a positive Dix–Hallpike manoeuvre.

#### Further reading

- Li J, Epley J. Benign paroxysmal positional vertigo. New York, NY: Medscape; 2016.
- South African Department of Health. Hospital level standard treatment guidelines and essential medicines list. Pretoria: National Department of Health; 2019.
- BMJ Learning. Vertigo – Dix-Hallpike manoeuvre from BMJ Learning [homepage on the Internet]. 2014. Available from: https://www.youtube.com/watch?v=8RYB2QlO1N4
- BMJ Learning. Vertigo – Epley manoeuvre from BMJ Learning [homepage on the Internet]. 2014. Available from: https://www.youtube.com/watch?v=jBzID5nVQjk

### Short answer question: The family physician’s role as care provider and capacity builder

You have recently been appointed as a senior family physician in a district hospital. Over the past few weeks, your junior clinician colleagues have frequently requested your opinion on the management of common dermatological conditions, most of which are referrals from the hospital catchment area. Some indicated that they had occasionally encountered emergency dermatological conditions at the hospital. You need to decide to improve clinical competency in the management of dermatological conditions in the district.

1. **Outline your plan to build capacity to address the identified need? (8 marks)**
  The approach to this question requires you to answer the following.
  - Who are the recipients? Establish staff complement of the health facilities referring to the hospital (healthcare team: medical officers, registrars, medical students, clinical associates and primary care nurses) (1 mark)
  - Who are the potential trainers? Identification of clinicians with the potential to train others and build a team of trainers with him or her. (1 mark)
  - What are the learning, knowledge and skill outcomes? (1 mark)
  - How will you organise the training? Arrange clinical training sessions, including workshops, bedside clinical demonstrations and external short courses on clinical dermatology. Arrange outreach training sessions for clinicians in the outlying community health centres (CHCs) and clinics. (3 marks)
  - How will you ensure the availability of resources to facilitate training? Insourced and outsourced human resources (clinical trainers), infrastructure (venue and equipment, including slide shows) and continuous professional development (CPD) accreditation. (1 mark)
  - So what impact has the training had and how can you improve your training in the future?
  - Get feedback and do an evaluation of the teaching and learning and the course content. (1 mark)
2. **How would you know if your plan to build capacity has worked? (4 marks)**
  - Assess knowledge at the end of training session. (1 mark)
  - Assess practice skills using a mini-CEX. (1 mark)
  - Audit – appropriateness of dermatological referrals, quality of care for dermatological conditions – chart-stimulated recall sessions. (1 mark)
  - Measure reduction of inappropriate referrals, from routinely collected data. (1 mark)
3. **What general points on supportive measures and management of dermatological emergencies would you emphasise whilst training clinicians? (Provide general points considering the following differential diagnosis: Stevens Johnson syndrome (SJS), toxic epidermal necrolysis (TENS), burns, pemphigoid/pemphigus.) (8 marks)**
  - Stop all potentially causative medicines. (1 mark)
  - If infection is suspected, take blood sample and skin lesion specimens for culture and sensitivity for possible initiation of antibiotic therapy. (1 mark)
  - Do not puncture bullae or vesicles. (1 mark)
  - Apply cold compresses and wet dressings. (1 mark)
  - Regularly supervise oral, genital and eye care to prevent adhesions and scarring. (1 mark)
  - Maintain fluid balance – beware of shock. (1 mark)
  - Provide nasogastric feeds if unable to eat. (1 mark)
  - Conditions needing referral to higher levels of care (e.g. intensive care unit [ICU]) require patient stabilisation before transfer. (1 mark)

**Total: 20 marks**

#### Further reading

- Mash R, Ogunbanjo G, Naidoo SS, Hellenberg D. The contribution of family physicians to district health services: A national position paper for South Africa. S Afr Fam Pract. 2015;57(3):54–61.
- South African Department of Health. Hospital level standard treatment guidelines and essential medicines list. Pretoria: National Department of Health; 2019.
- De Villiers M. Chapter 174: How to plan and implement a teaching activity or a continuing professional development meeting. In: Mash B, Blitz J, editors. South African Family Practice Manual. 3rd ed. Hatfield, Pretoria: Van Schaik, 2015; p. 594–596.
- De Villiers M, Van Schalkwyk S. Chapter 177: How to facilitate small-group learning. In: Mash B, Blitz J, editors. South African Family Practice Manual. 3rd ed. Hatfield, Pretoria: Van Schaik, 2015; p. 603–605.
- De Villiers M, Couper I, Coetzee F. Running effective workshops – Running small groups [homepage on the Internet]. Podcast. South African Association of Health Educationalists. 2020 [cited 2020 Sep 29]. Available from: http://saahe.org.za/category/podcast/

### Critical appraisal of quantitative research

#### Critical appraisal of research

Read the accompanying article carefully and then answer the following questions (total 40 marks). As far as possible, use your own words. Do not copy out chunks from the article. Be guided by the allocation of marks with respect to the length of your responses.

Desalew A, Feto Gelano T, Semahegn A, Geda B, Ali T. Childhood hearing impairment and its associated factors in sub-Saharan Africa in the 21st century: A systematic review and meta-analysis. SAGE Open Med [serial online]. 2020 [cited 2020 September 15];8:1–11. Available from: https://journals.sagepub.com/doi/full/10.1177/2050312120919240

1. What research question did the authors attempt to answer in this study? Comment on whether this was a clearly focused question in terms of the PICO framework (patient group, patient problem or population of interest, intervention or issue of interest, comparison intervention of interest, primary outcome of interest). (5 marks)
2. Considering the background section in this article, identify two sentences or phrases that best reflect the authors’ starting point, from which the rationale for the research is further explained and/or elaborated (more than one correct answer possible). (2 marks)
3. Was the search for relevant primary studies to include in the review detailed and exhaustive? (5 marks)
4. Were the criteria and process used to select primary studies for inclusion in the review appropriate? (5 marks)
5. Were the included primary studies of high methodological quality? In other words, did the authors do enough to assess the quality of the included studies? (3 marks)
6. Were the results similar from study to study in the included primary studies? In other words, appraise the results with specific reference to the forest plots and heterogeneity. (4 marks)
7. What are the overall results of the review? (6 marks)
8. Were the specific directives for new research appropriate? (4 marks)
9. Discuss the value of the study findings for your own practice. (6 marks)

**(Total: 40 marks)**

#### Suggested answers

**FIGURE 2:**
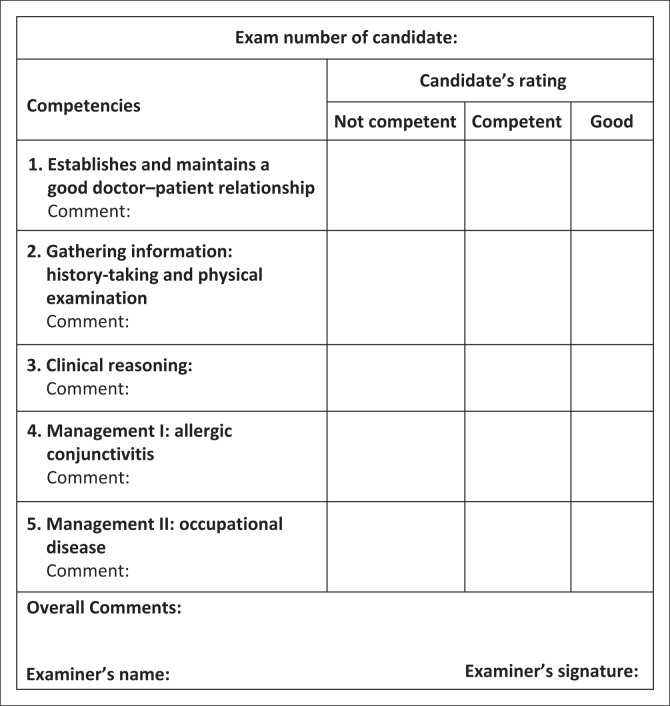
Marking template for consultation station.

1. **What research question did the authors attempt to answer in this study? Comment on whether this was a clearly focused question in terms of the PICO (patient group, patient problem or population of interest, intervention or issue of interest, comparison intervention of interest, primary outcome of interest) framework. (5 marks)**
  The authors attempted to determine the pooled prevalence of childhood hearing impairment and its associated factors in sub-Saharan Africa (SSA).
  The PICO framework is generally used to help frame or focus the research question and subsequent search for relevant evidence. The framework may be tailored to the research question type (treatment, prevention, diagnosis, prognosis or aetiology) or study design (quantitative compared to qualitative).
  Using the PICO framework for this study, the population of interest (P) would be children in SSA; the issue of interest (I): the pooled prevalence of childhood hearing impairment and its associated factors. Although there is no explicit comparison intervention of interest, the context (C) is that of SSA. The outcome of interest (O) would be the magnitude of childhood hearing impairment and its associated factors.
  This systematic review, therefore, aimed to answer a broad question, which covers the domains from the PICO framework (see search strings described in the ‘Data source and search strategies’ section: population, outcome, study design and location); one wonders whether a scoping review would have been more appropriate given the broad focus of the question. Alternatively, a systematic review question with a narrower focus would have been more appropriate, for instance, by qualifying whether the context is urban versus rural, community based versus facility based, primary care versus specialised care; or differentiating between pre-school and school children.
2. **Considering the introduction section in this article, identify two sentences or phrases that best reflect the authors’ starting point, from which the rationale for the research is further explained and/or elaborated (more than one correct answer possible). (2 marks)**
  Potential options include:
  - Hearing impairment is a significant cause of disability worldwide and more than two-thirds of the population with hearing impairment live in developing countries.
  - The burden of hearing impairment is more in developing countries, specifically sub-Saharan Africa (SSA), where the majority of children with significant hearing problems are living.
  - The sense of hearing is fundamental to facilitate communication and foster social interaction. In children, disabling hearing impairment impedes speech and language development and affects children’s educational and vocational attainment.
  - Without suitable interventions, hearing impairment is a barrier to both education and social integration. These consequences can be reduced by early detection with appropriate audiological and speech interventions.
  - Whilst hearing aid is used to reduce the burden of hearing impairment in high-income countries, there is little evidence of their use in developing countries. However, identifying the leading causes of hearing impairment and implementing preventive action could reduce the hearing-related problems in developing countries.
  - Despite the ratification of existing laws and policies on disability by many countries and some progress made in terms of legislative and policy reform, the realities for children with disabilities have not yet changed mainly because of poverty and the lack of human resources. As a result, the number of children with hearing disabilities and those living with disabilities are grossly underestimated.
  - This demonstrates the demand for a comprehensive analysis of the magnitude of hearing impairment to inform policymakers, programme planners, service providers, advocators and concerned stakeholders to place more emphasis on childhood hearing impairment in developing countries.
3. **Was the search for relevant primary studies to include in the review detailed and exhaustive? (5 marks)**
  This question may be answered by appraising the methods section of the manuscript, which describes if the ‘best sort of studies’ to address the research question and with the appropriate study design were selected. A good systematic review should describe the totality of the available data (published and unpublished) and a multifaceted search approach should be used.
  For this study, the concern regarding the scope of the question was mentioned here; ideally, a clear and focused question should guide the search. The search strategy was informed by search strings linked keyword relating to population, outcome, study design and location. A literature search was carried out on the main electronic databases and indexing platforms (such as PubMed, Medline, EBSCOhost and African Journals Online). Other relevant sources, such as Google Scholar and WHO websites, were also used to search studies. The authors state that, ‘both published and unpublished studies from 2000 to 2018, which were written in the English language and fulfilled all other criteria were included in the systematic review’. The authors did not specify how they obtained the unpublished studies; admittedly, the flow diagram in Figure 1 mentioned that 80 studies identified through other sources were identified (unpublished sources may include personal files of experts, meeting abstracts or conference proceedings and theses). However, the strategy to identify these other sources was not described in this article but may have specified in the study protocol registered on the International Prospective Register of Systematic Reviews database (PROSPERO). The language bias towards English may have excluded important studies from francophone SSA countries (useful research published in other languages may have been missed; this bias also skews the population to whom the research findings may be applied).
4. **Were the criteria and process used to select primary studies for inclusion in the review appropriate? (5 marks)**
  The authors stated that, ‘all studies with the primary objective to determine the prevalence of hearing impairments and its associated factors amongst children in SSA were considered’. The inclusion and exclusion criteria are described in the methods section and revolve around study type (observational, quantitative vs. qualitative), study setting (community and facility based), prevalence of hearing impairment with or without associated factors, English language, as well as time period (2000–2018: this arbitrary time period choice was not clarified).
  One may conclude that the criteria used to select primary studies for inclusion in the review were mostly appropriate. There is a concern around the exclusion of non-English publications as well as studies before 2000 (presumably, such studies would be too dated for inclusion, but this choice was not discussed by the authors).
  An important part of this screening and selection process is the consideration of publication bias in the meta-analysis. Funnel plots were performed in this study but only mentioned in the text and no results of the Egger’s tests were presented. The assessment of the symmetry of the funnel plots regarding publication bias is, therefore, not possible.
  Figure 1 displays the flow diagram that illustrates how the studies were screened in terms of eligibility criteria. Ultimately 26 studies were included in a systematic review. The authors described the process of screening in the methods section. It must be noted that the two authors independently screened the studies based on the preset inclusion criteria. Studies that fulfilled the eligibility criteria based on the titles and abstracts were retrieved for full-text screening. The full-text screenings were carried out by the same two independent authors and two additional authors were consulted to resolve any disagreements regarding the selection process as the flow diagram was adapted from the Preferred Reporting Items for Systematic Reviews and Meta-Analyses (PRISMA) reporting guidelines.
5. **Were the included primary studies of high methodological quality? In other words, did the authors do enough to assess the quality of the included studies? (3 marks)**
  In the methods section, the critical appraisal of the studies is described. The studies were evaluated to determine the validity of their findings. A critical appraisal checklist for observational studies designed by the Joanna Briggs Institute (JBI) was used to determine the methodological robustness and validity. The scores of the two authors (who also screened the studies) were used in consultation with the two additional authors to determine the final selection.
  Particular attention was given to clear statements regarding the objective of the studies, sampling techniques, precision of measurement of outcomes of interest and exposure variables, as well as documentation of sources of bias or confounding.
  It is, therefore, reasonable to conclude that the authors aimed to assess the quality of the included studies using a structured manner. The JBI critical appraisal checklist was used to determine which studies to include in the systematic review and meta-analysis. Studies with positive responses greater than half of the number of checklists (i.e. a score of 5 or more) were included.
6. **Were the results similar from study to study in the included primary studies? In other words, appraise the results with specific reference to the forest plots and heterogeneity. (4 marks)**
  The 26 studies selected for the systematic review and meta-analysis are presented in Table 1 in the article. The description of each study includes the author, year and country, the study design, primary interest, target population, sample size, diagnostic method, normality criterion (for determining hearing loss) as well as key findings and risk factors. This allows the reader to have an overview of the nature of the studies included in the review. The study characteristics of the primary studies included in the review are described in the results section: 21 studies were cross-sectional, three studies were case-control and the remaining studies were prospective cohort. All studies were conducted in the predetermined time range and were written in English. The sample sizes in the primary studies ranged considerably from 94 to 21 572. From the data in Table 1, it must be noted that the study settings (country), target population, diagnostic method and normality criterion varied considerably.
  The visual inspection of Table 1 is complemented by the statistical tests used in the meta-analysis of the combined findings of the primary studies. The presence of statistical heterogeneity was assessed in this study by using the Cochrane’s Q test (the statistical test most used is a variant of the Chi-square [***χ***^2^] test, namely, the ***χ***^2^ statistic for heterogeneity). In this meta-analysis, the levels of heterogeneity amongst the studies were quantified using the *I*^2^ statistics; the authors state in the methods section that substantial heterogeneity was assumed if the *I*^2^ value was ≥ 60%. The forest plots denoting the prevalence of hearing impairment in SSA are shown in Figures 2–4. Forest plots summarise all the studies according to a common statistic (in this case, the prevalence of hearing impairment) and display them on a single axis, which allows comparison of the studies and the quality of the final result. The diamond shape represents the point estimate and confidence intervals (CIs), which summarise the average of all the individual studies. In terms of heterogeneity, one would expect that different studies looking at the same question through similar methods should show consistent results across all the studies; unfortunately, this is rarely the case, as many factors affect the results of studies, such as bias or data collection problems. In all three figures presented in this review, the *I*^2^ values are around 98% – 99%, which denote significant heterogeneity. This heterogeneity was expected given the wide range of characteristics of the studies included in the review, but regrettably means that the combined findings of the studies are inconsistent and make any conclusions drawn from these forest plots questionable.
7. **What are the overall results of the review? (6 marks)**
  The results section of the article describes the critical analysis of the 26 articles used in the review under the following headers: the pooled prevalence of hearing impairment and the risk factors of hearing impairments. From a critical appraisal perspective, the reader should be clear about the ‘bottom line’ of the review’s results. The discussion section starts off with a summative statement about the social and scientific value of identifying the prevalence of hearing impairment and its associated factors , as well as about the authors’ desire to make recommendations to prevent hearing impairment.
  The key findings were that the pooled prevalence of hearing impairment was 10% (95% CI: 9% – 11%) and several factors were associated with hearing impairment amongst SSA children (chronic suppurative otitis media, impacted cerumen, advanced stage of human immunodeficiency virus [HIV], tuberculosis infection and age). The authors performed a subgroup analysis based on population characteristics included in the study. The pooled prevalence of hearing impairment for school or community-based children was 6% (95% CI: 5% – 7%). In addition, the pooled prevalence of hearing impairment for children with comorbidities was 23% (95% CI: 15% – 31%).
  However, there were several limitations related to the clinical heterogeneity of the studies included, such as different methods of identifying hearing impairment (including auditory threshold and otoscopy, automated pure tone audiometry, and World Health Organization [WHO] or United Nations Children’s Fund [UNICEF] questions-based interviews with parents), the different cut values or decibel thresholds used to discern between normality and hearing impairment (ranging from 20 to 40 decibels), as well as the range of population groups included in the primary studies (school-based or community children, different age ranges within age groups and children living with comorbidities such as HIV and tuberculosis infection).
  The authors described the study limitations at the end of the discussion section: studies included were published in English only, were observational in nature and had high heterogeneity. This review was not powered to formally assess associations of hearing impairment.
  Although not described by the authors, the reader may wonder whether these 26 primary studies were representative of SSA, as only 10 countries were included. A breakdown of the primary studies by country is shown in the table here and illustrates the unequal distribution of the primary study sample across the SSA region (*Table 1 in the current article discussed* [Desalew et al. 2020]*is not part of the model answer and only serves to illustrate the point made regarding the skewed geographic distribution of the primary studies*).
8. **Were the specific directives for new research appropriate? (4 marks)**
  Yes, the authors recommend in the conclusion section that a well-designed epidemiological study should be conducted in a more representative population using standardised definitions of hearing impairment and objective methods for case ascertainment. In addition, the authors recommend that the diagnosis modality should be standardised for studies in SSA and other developing countries. These research directives only reflect epidemiological approaches; it would have been good to also specify other types of primary care research, such as applied research and participatory research, which engage various stakeholders and employ implementation science principles.
  Furthermore, the authors state in the opening paragraph of the discussion section that this systematic review summarised up-to-date empirical evidence and identified key areas of action. However, these key areas of action are limited and would have been enhanced by a more detailed description of activities for policymakers, researchers and practitioners, as well as parents and community members. Admittedly, the authors recommended regular community and school-based screening activities for early detection and necessary intervention programmes designed by concerned stakeholders on childhood hearing impairment. However, this general statement would have been enhanced by specifying the research and policy strategies at local, national and regional levels.
9. **Discuss the value of the study findings for your own practice. (6 marks)**
  The relevance, education, applicability, discrimination, evaluation and reaction (READER) format may be used to answer this question:
  - Relevance – does it relevant to family medicine and primary care?
  - Education – does it challenge existing knowledge or thinking?
  - Applicability – are the results applicable to my practice?
  - Discrimination – is the study scientifically valid enough?
  - Evaluation – given the above points, how would I score or evaluate the usefulness of this study to my practice?
  - Reaction – what will I do with the study’s findings?
  The answer may be a subjective response but should be one that demonstrates a reflection on the possible changes within the student’s practice within the South African public healthcare system. It is acceptable for the student to suggest how his or her practice might change, within other scenarios after graduation (e.g. general private practice). The reflection on whether all important outcomes were considered is therefore dependent on the reader’s own perspective (is there other information you would have liked to see?).
  A model answer could be written from the perspective of the family physician employed in the district health system: this systematic review is relevant to the African primary care context, as child and adolescent health form core aspects of the primary healthcare approach. The intended target audience includes ‘policymakers, programme planners, service providers, advocators as well as concerned stakeholders to place more emphasis on childhood hearing impairment in developing countries’, which makes the review also relevant to the clinician at the coalface. The issues highlighted by the systematic review may resonate with the challenges experienced in primary healthcare, as family physicians and their healthcare teams need to provide a comprehensive service to their patients, families and communities, including addressing issues of hearing impairment in children, which may affect their growth and development potential. In terms of discrimination, the significant heterogeneity means that the combined findings of the studies are inconsistent and make any conclusions drawn questionable. The role of team-based care and linkages between generalist primary care providers, school health teams and specialised audiology providers were not mentioned specifically. An integrated service model should look at the interface between the curative components of the local health service and the preventative, screening and health promotion aspects of a sound primary healthcare service. The study may be discussed with the local and district management team and be used as a basis for improving the local health service to ensure better care coordination and inter-sectoral engagement with the education and municipal services in the spirit of community-orientated primary care. Childhood hearing screening services using standardised cut values should form part of the team-based approach to provide evidence-informed healthcare.

**TABLE 1:** Distribution of the primary study sample across the sub-Saharan African region.

Country	No. of primary care studies
Cameron	1
Ethiopia	1
Kenya	1
Malawi	4
Mozambique	1
Nigeria	5
Senegal	1
South Africa	6
Uganda	4
Zimbabwe	2
Total	26

*Source:* Desalew A, Feto Gelano T, Semahegn A, Geda B, Ali T. Childhood hearing impairment and its associated factors in sub-Saharan Africa in the 21st century: A systematic review and meta-analysis. SAGE Open Med [serial online]. 2020;8:1 -11. Available from: https://journals.sagepub.com/doi/full/10.1177/2050312120919240

#### Further reading

- Naude C, Young T. How to search and critically appraise the literature. In Goodyear-Smith F, Mash B, editors. How to do primary care research. 1st ed. Boca Raton, FL: CRC Press, 2019; p. 135–146.
- Joannabriggs.org. Critical Appraisal Tools – JBI [homepage on the Internet]. 2020 [cited 2020 Sep 26]. Available from: https://joannabriggs.org/critical-appraisal-tools
- The Critical Appraisals Skills Programme (CASP). 2020. CASP checklists [homepage on the Internet]. [cited 2020 Sep 26]. Available from: https://casp-uk.net/casp-tools-checklists/
- Critical Appraisal Tools. 2020. Oxford Centre for Evidence-Based Medicine [homepage on the Internet]. [cited 2020 Sep 26]. Available from: https://www.cebm.ox.ac.uk/resources/ebm-tools/critical-appraisal-tools
- Resources. 2020. The evidence-based healthcare programme [homepage on the Internet]. [cited 2020 Sep 26]. Available from: http://www.ebhc.uct.ac.za/resources-33
- Ross A, Mash B. African primary care research: Reviewing the literature. Afr J Prim Health Care Fam Med. 2014;6(1):1–4. https://doi.org/10.4102/phcfm.v6i1.584
- Taylor P, Hussain JA, Gadoud A. How to appraise a systematic review. Br J Hosp Med. 2013;74(6):331–334. https://doi.org/10.12968/hmed.2013.74.6.331
- Greenhalgh T. Chapter 9: Papers that summarise other papers (systematic reviews and meta-analyses). In: Greenhalgh T, editor. How to read a paper: The basics of evidence-based medicine. 5th ed. Chichester, West Sussex: John Wiley & Sons, 2014; p. 116–134.

### Objectively structured clinical examination scenario

#### Objective of station

This station tests the candidate’s ability to:

- manage a patient with allergic conjunctivitis
- manage an occupational disease.

#### Type of station

Simulated consultation.

#### Resource list

- Role player – patient: male/female in their 30s.

### Instructions for candidate

#### History/context

You are the family physician working in a CHC.

You are asked by the triage nurse to consult with the following unbooked patient.

Please consult with this patient and develop a comprehensive management plan.

You do not need to perform a physical examination.

Physical examination findings and investigations will be provided to you on request.

#### Instructions for the examiner

**Objectives:** This station tests the candidate’s ability to:

- manage a patient with allergic conjunctivitis
- manage an occupational disease.

This is a simulated consultation station in which the candidate has 14 min.

Familiarise yourself with the assessor guidelines, which details the required responses expected from the candidate.

No marks are allocated. In the mark sheet, tick off one of the three responses for each of the competencies listed. Make sure you are clear on what the criteria are for judging a candidate’s competence in each area.

Provide the following information to the candidate when requested: *clinical findings*

Please switch off your cell phone.

Please **do not** prompt the student.

Please ensure that the station remains tidy and is reset between candidates.

This station is 15-min long. The candidate has 14 min, and you have 1 min between candidates to complete the mark sheet (Figure 2) and prepare the station.

- Adams S, Jeebhay M. Chapter 75: How to claim compensation for work-related injuries or diseases. In: Mash B, Blitz J, editors. South African Family Practice. 2nd ed. Pretoria: Van Schaik, 2006; 542–548.
- National Department of Health. Ch 18.1.1: Allergic conjunctivitis. In: Essential medicine list for primary care. 2018.

### Guidance for examiner: Some general descriptors of competencies

**Working definition of competent performance:** the candidate *effectively completes the task* within the allotted time in a manner that *maintains patient safety*, even though the execution may not be efficient and well structured.

- **Establishes a good doctor–patient relationship**
  Shows genuine respect, compassion, sensitivity, rapport and empathy; establishes trust; and attends to patient’s comfort. Acknowledge patient’s discomfort and the anxiety related to ongoing physical symptoms.
  A **competent candidate** acts within the ethical framework (respects autonomy, justice, non-maleficence and beneficence). In addition, the **good candidate** displays empathy and compassion.
- **Gathering information: history and physical examination**
  The **competent candidate** gathers sufficient information to identify current medical issues (*allergic conjunctivitis and occupational exposure*) and identify any ongoing biopsychosocial risks. In addition, the **good candidate** explores the patient’s experience (*decreased quality of life and conflict with manager*), fears and expectations (*may need to change jobs*) and health-seeking behaviour and identifies opportunities for health promotion (*occupational health promotion*).
- **Clinical reasoning**
  The **competent candidate** uses available evidence to make the correct working diagnosis (*allergic conjunctivitis because of occupational chemical exposure*). The good candidate is able to make a comprehensive three-stage assessment (*occupational allergic conjunctivitis, impact on quality of life [*QoL*], conflict with manager and need for advocacy to Occupational Health and Safety Act*) or uses a standardised model (e.g. Stott’s model).
- **Management I: allergic conjunctivitis**
  The **competent candidate** uses current evidence-based guidelines to develop a management plan. In addition, the **good candidate** develops a comprehensive plan within a biopsychosocial approach and is risk aware (*advises on caution using eye drops, and when to refer to a specialist*).
- **Management II: occupational disease**
  The **competent candidate** uses current evidence-based guidelines to develop a management plan (i.e. *informs patient of their rights and mentions the Compensation for Occupational Injuries and Diseases Act [COIDA No 130 of 1993] process*). In addition, the **good candidate** develops a comprehensive plan within a biopsychosocial approach (i.e. *displays good working knowledge of the OHSA, COIDA processes and how this applies to this situation*).

### Additional guidance for examiner

#### Acute management of allergic conjunctivitis

- Identification of the allergen/irritant
- Remove contact lenses
- First-line treatment: topical oxymetazoline eye drops – maximum 7 days
- Can add cetirizine 10 mg daily.

#### Occupational disease

- OHSA comes into effect when an illness/injury is incurred within the normal execution of the job.
- Employee should report to employer as soon as an injury/illness occurs.
- Employer must report to the Department of Labour within 14 days.
- Medical practitioner completes First Medical Report – no need to wait for employer to complete documentation – include a report and recommendations and need for specialist intervention:
  ▪ Involve family and spiritual counsellor
  ▪ Multi-disciplinary team (MDT) approach
  ▪ Advanced-care planning.

### Examination findings and investigations

Add the relevant details for the examiner – examiner ***should NOT*** show all the examination findings to the candidate, but should respond to specific questions being asked.

General examination

- All vitals normal

Systematic examination

- Normal

Eyes

- Watery, inflames conjunctivae
- Iris, pupil normal
- Eye movements normal
- Visual acuity normal

#### Role play: Instructions for actor

**Appearance and behaviour:** Neat, well-groomed adult. Wearing sunglasses because of light sensitivity.

**Opening statement:** ‘Dr, I have this problem with my eyes, itchy and teary all the time, and I can’t handle the light’.

### History

#### Open responses: Freely tell the doctor

- The problem started about 1 week ago and progressively getting worse.
- You think it is caused by a new chemical that was introduced 2 weeks ago – you work in a printing company.

#### Closed responses

Only tell the doctor if asked …

- Over the weekend, you had minimal symptoms.
- It usually starts when you walk into the office and gets worse during the day.
- You have no other symptoms.
- It has affected your quality of life and you cannot drive, work on your computer and it is very uncomfortable.

Ideas, concerns and expectations:

- You have discussed this with your boss – he does not believe you – no one else is complaining of the same problem.
- You are considering approaching the union representative, but need more information.

Medical history:

- Besides this problem, nothing else

Family and social history:

- You have been working in this company for 4 years, with no issues before. But management is very profit orientated.

